# Human AGEs: an interactive spatio-temporal visualization and database of human archeogenomics

**DOI:** 10.1093/nar/gkad428

**Published:** 2023-05-22

**Authors:** Lukasz Ciecierski, Ireneusz Stolarek, Marek Figlerowicz

**Affiliations:** Institute of Bioorganic Chemistry, Polish Academy of Sciences, Poznan, Poland; Institute of Bioorganic Chemistry, Polish Academy of Sciences, Poznan, Poland; Institute of Bioorganic Chemistry, Polish Academy of Sciences, Poznan, Poland

## Abstract

Archeogenomics is a rapidly growing interdisciplinary research field driven by the development of techniques that enable the acquisition and analysis of ancient DNA (aDNA). Recent advances in aDNA studies have contributed significantly to increasing our understanding of the natural history of humans. One of the most significant challenges facing archeogenomics is the integration of highly heterogeneous genomic, archeological, and anthropological data and their comprehensive analysis, considering changes that occur in time and space. Only this complex approach can explain the relationship between past populations in the context of migration or cultural development. To address these challenges, we developed a Human AGEs web server. It focuses on creating comprehensive spatiotemporal visualizations of genomic, archeogenomic, and archeological information, which can be provided by the user or loaded from a graph database. The interactive map application at the center of Human AGEs can display multiple layers of data in various forms, such as bubble charts, pie charts, heatmaps, or tag clouds. These visualizations can be modified using various clustering, filtering, and styling options, and the map state can be exported to a high-resolution image or saved as a session file for later use. Human AGEs, along with their tutorial, are accessible at https://archeogenomics.eu/.

## Graphical Abstract

**Graphical Abstract:**
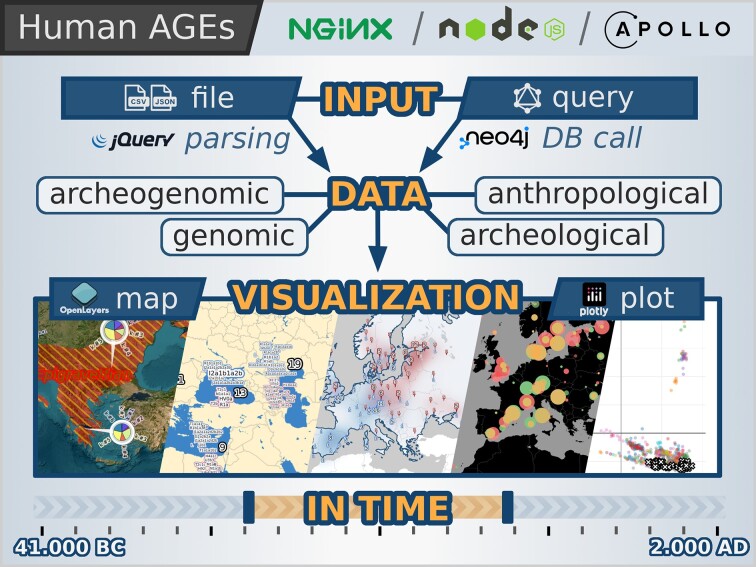

## INTRODUCTION

During the last decade, ancient DNA (aDNA) research has significantly improved our understanding of the biological history of humans. Archeogenomics sheds light on a range of issues related to the origins and dispersal of modern humans across the globe, as well as to the genetic diversity and adaptations of extinct hominin species. With the development of next-generation sequencing technologies, the amount of aDNA data has increased exponentially, opening new avenues of research and leading to significant discoveries.

The rapid expansion of data has shifted the character of aDNA studies from human genetics, mainly using fragments of mitochondrial DNA, to population genomics fueled by thousands of genome-wide datasets, nearly complete mitochondrial genome assemblies, and detailed descriptions of Y-chromosome lineages (2–6). Furthermore, it is becoming increasingly clear that the archeological context of the studied human remains is also of high importance in the field.

Collectively, these results make the human archeogenomic data extremely complex. Their transdisciplinary character means that they should be accessible to representatives of both life sciences and humanists. However, despite the immense growth of the aDNA field and the huge interest in society, there is no web server that provides a convenient spatiotemporal analysis of archeogenomic data.

Because of this unsatisfied demand, we present the Human ArcheoGEnomics (Human AGEs) web server for archeogenomic data visualization and interactive analysis.

## MATERIALS AND METHODS

### Web server

Human AGEs is hosted on a Linux-based Nginx HTTP server. We used Nodejs web application server for back-end development and the Pug.js template engine, SASS stylesheet, and JavaScript programming languages for front-end development. To create the main visualization features, we applied OpenLayers geographic and Plotly charting libraries. Human AGEs loading time was reduced using static content hosting Content Delivery Network (CDN) services. Moreover, we applied Webpack module bundler to optimize the website files and increase compatibility with older browsers. More information regarding the software is provided in the Supplementary Data (Supplementary Table S1; Supplementary Table S2).

### Data sources

The Human AGEs web server hosts several publicly available genomic and archeogenomic datasets. These include the Allen Ancient DNA Resource (AADR) v54.1 (https://reich.hms.harvard.edu/allen-ancient-dna-resource-aadr-downloadable-genotypes-present-day-and-ancient-dna-data", version 54.1.p1) and EMPOP v4/R13 dataset (1) (https://empop.online/, version 4). AADR data consists of genome-wide data across 1.2M single-nucleotide polymorphism (SNP) positions, Y-chromosome, and mitochondrial genome haplogroup assignments. The EMPOP dataset consists of the present-day mitochondrial genome haplogroup assignments.

Additionally, to showcase Human AGEs visualization capabilities, we hosted precomputed PCA and UMAP embeddings of AADR samples as well as the proportions of admixture components calculated from *k* = 2 to *k* = 13 (Supplementary Methods). We also provide a source of archeological data in the form of manually-curated archeological culture regions (*N* = 109), which are available for visualization on the interactive map.

### Graph database

Published genomic data sources were incorporated into our graph database powered by Neo4j. The database was populated using Neo4j import methods and custom-made scripts. To facilitate a connection between the web application and graph database, we used Apollo server. To enable graph query calls and graph data exchange between the client and the Human AGEs server, we used GraphQL API.

## RESULTS

### General description of the human AGEs server

Human AGEs is a web server dedicated to the interactive visualization of archeogenomic data. Its two main features are (i) an interactive spatio-temporal map and (ii) a graph database. The Human AGEs server is publicly available and free to use. It does not depend on cookie files and does not block any content by requiring user login credentials. The web server was tested on Firefox v. 110.0 and Chrome v. 110.0 browsers on Windows and Linux systems.

### Input and output data

**Figure 1.**
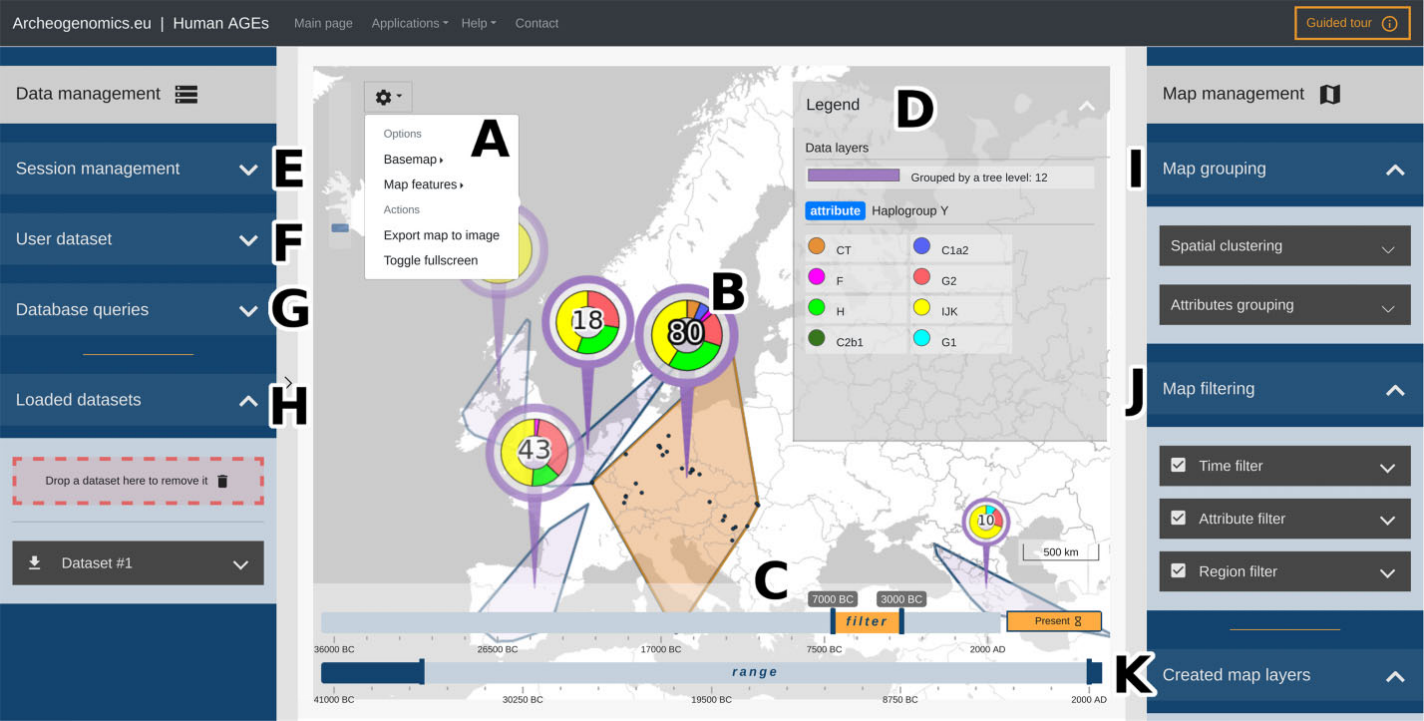
Interactive map application interface. (**A**) The toolkit menu contains map appearance options and an export-to-image feature. (**B**) Samples cluster map marker. (**C**) The timeline filter and range sliders are used to navigate through data in the dimension of time. (**D**) Dynamic legend shows currently visible map layers and style applied to data attributes. (**E**) Section containing map state import and export options. There is also an option to load example sessions. (**F**) Section providing options to load user dataset. (**G**) Section providing graph database query builder, a text box containing the query JSON string, and an option to call the database. (**H**) Section listing loaded datasets together with their download and sampling options. The datasets can be removed by drag-and-drop interaction. (**I**) Section consisting of spatial clustering and attributes grouping features. (**J**) Section with options of time, attribute and region filters. (**K**) List of created map layers. It is possible to create new layers, clone existing ones, or remove them.

To load present-day genomic or archeogenomic data for visualization, users can either upload their own input files (Figure 1F) or use one of the data sources stored in our graph database (Figure 1G). In the first case, the user can upload an input file in CSV or JSON format. In addition to mandatory sample ID and coordinates, it is possible to use one of the available data types to include information regarding the Y chromosome (Y-)DNA and mitochondrial (mt)DNA haplogroup assignments, results of admixture analysis, UMAP or PCA embeddings, and custom data attributes (Supplementary Table S3 and Supplementary Table S4).

In the second case, users can load either a present-day genomic or archeogenomic dataset by choosing one of the data sources available in the Human AGEs graph database. Its content can be limited by the multiple filters defined in our query builder (Supplementary Figure S1A). The data source names and filters together produce a JSON query call, which is a unique identifier of the dataset and can be safely used as a reference (Supplementary Figure S1B).

All loaded datasets can be downloaded using the download button of the dataset (Figure 1H). With the map toolbox (Figure 1A), the current map view can be saved as an image in one of the following formats: JPG, PNG and PDF.

### Session file

Session is a powerful utility that can be treated as both the input and output of the Human AGEs web server. This enables users to save their entire interactive map state to a human-readable JSON session file (Figure 1E). This file contains the map state and all previously set configurations (that is, data filters and attribute colors), which can later be shared and restored.

### Interactive map

The interactive map application is the main feature of the Human AGEs server. Samples are represented on the map as markers that can be hovered, selected, or dragged (Figure 1B). Double-clicking a marker will open its details window listing all selected samples and their attributes (that is, admixture proportions, haplogroup assignments, visualization on UMAP or PCA plot). The map is supported by an interactive timeline, dynamic legend, and other useful features, such as zoom control, map scale and title. The timeline consists of two draggable intervals (Figure 1C). The first is the time filter, which controls the data sample visibility owing to their dating. The second is the time range, which sets a lower and upper bound for the filter values.

The legend displays the style of the visible map layers, data attributes and archeological cultures (Figure 1D). The style is represented by a symbol on the left of a name, and it can be modified in the menu by clicking. The legend is dynamic; it updates with changes made to the data or map, for example, by limiting lists of attributes and regions to only those visible in a current map view. Finally, the map appearance can be changed using options in the map toolbox. Available modifications include basemap type and style, map feature visibility, and appearance.

### Data clustering

The aim of the clustering feature is to generalize the visualization by reducing data resolution. This can be achieved in two ways: with spatial clustering aggregating samples by their position, or with attribute clustering, which allows the creation of groups of attribute values (Figure 1I).

Spatial clustering operates in two distinct modes that can be combined. First, it can group samples based on their absolute (kilometers) or relative (pixels) distance to another randomly chosen sample serving as a central point. Second, it assigns samples to the same cluster if they lie within the same region. For this, we provide different regional sets of geographical, political, and archeological types. The latter includes archeological cultures and, unlike the others, the territory of these regions changes dynamically with a time filter.

Attribute clustering can be performed manually or automatically. Manual clustering requires the user to select the attribute values from a dedicated browser, and add them to a new or existing group by providing its name. Automatic clustering options are available only for tree-based attributes such as haplogroups. They allow the creation of a group of ancestor or descendant haplogroups in relation to a given haplogroup, group more specific haplogroups to their ancestors at a given cutoff tree level, and assign haplogroups to a group named after their root words of a given length. All the created attribute groups can be modified, expanded, removed, or filtered at later stages through attribute filtering options.

### Data filtering

Filtering aims to simplify the visualization by narrowing its scope. This can be achieved by filtering objects by time, attribute values or region names (Figure 1J).

Time filtering is mostly facilitated by the map timeline; however, exact filter interval numbers can be input manually for better precision. In addition, this filter is supported by options concerning performance optimization and the object visibility fading function to enhance visualization customization.

Attribute filtering can be simultaneously applied to multiple attributes at the same time. Its main feature is filtering samples by the attribute's value name or presence. Moreover, some attribute types have unique sets of options. Tree-based attribute values (that is, haplogroup assignment) can be filtered by their distance from the root of the tree. Admixture attribute filters can discard samples based on the proportions of particular admixture components.

The last is region filtering, which can exclude already visible regions and their clustered samples according to the name of a region.

### Data styling

Dataset samples can be visualized using four types of map layers: (i–ii) the points and heatmap types are suitable for general presentation of the distribution of the samples; (iii–iv) the piecharts and tag clouds allow additional visualization of the content and frequencies of the given attribute's values present in the samples (Figure 1K; Supplementary Figure S2).

Map layers have a wide range of sample cluster styling options, including cluster position offset and rotation; cluster size, which is dependent on sample count; filtering out low-frequency or missing values; and layer-specific options concerning piechart labels or tag clouds’ word spacing. Map layers and attributes are uniquely indicated by a single adjustable color, or in the case of heatmaps, the color gradient. Additionally, archeological cultures can be styled using colored patterns.

### Example use case

Out of Africa is one of the most recognized hypotheses regarding human genesis and dispersal. Although the introduction of the latest DNA evidence caused some disagreements concerning the exact time, migration paths, and potential interactions with archaic hominins, the main aspects of the hypothesis remain valid: approximately 70 000 years ago, East African hunter-gatherers spread through the Arabian Peninsula, populating the rest of the globe. It was proposed that the African origin of modern humans can be inferred from the genetic mtDNA diversity of the present-day population. To verify this, we visualized contemporary mt haplogroup distribution using EMPOP samples clustered in continent regions. Sample mtDNA haplogroups were assigned to one of the ancestral groups in sequence: L3, L4, L6, L2, L5, L1 and L0 (Supplementary Figure S3). Additional examples are available at: https://archeogenomics.eu/en/examples.

## DISCUSSION

The explosion of archeogenomic studies is of significant interest in the biological history of humans and has resulted in numerous difficulties with presenting and analyzing the data due to their size, complexity, and interdisciplinary character.

To the best of our knowledge, there is no web server dedicated to customizable archeogenomic visualizations that addresses uniparental haplogroups and genome-wide data in anthropological and archeological contexts. The most similar tools include AmtDB (7), EMPOP and AncientGenomes (https://ancientgenomes.com/). The first one is dedicated to aDNA data; however, it focuses only on mtDNA, and features are limited to simple data filtering and visualizing sample locations on a basic map, which cannot be customized. However, the database has the option of downloading selected entities. It also allows for time filtering and provides a wide range of sample metadata that serve as the attributes to be filtered.

The EMPOP database has a large suite of analysis tools, phylogenetic trees, and network construction. A useful feature also includes the visualization of the selected mtDNA haplogroup frequency in the form of a heatmap. However, it is also dedicated only to mtDNA and only to present-day samples.

The AncientGenomes is another web application dedicated to spatio-temporal aDNA data presentation. It contains both mtDNA and Y-DNA haplogroups for ancient samples as well as spatial distribution of archeological cultures and ancient language families. It also dynamically presents information about major historical events, which is a valuable educational asset. The application, however, is mainly presentational, as it does not provide any attribute filtering options, the samples can only be presented as points and there are no options regarding their customization apart from possibility to toggle event, cultural or linguistic layers on and off.

Additional resources to study aDNA have been created by the scientific community members but yet unpublished. The features of Human AGEs webserver and other similar web applications were compared in more detail in Supplementary Data (Supplementary Table S5).

Therefore, Human AGEs is the first comprehensive web server dedicated to aDNA. It provides a wide range of interactive tools to filter and analyze various types of data with respect to their spatial or temporal distribution.

### Limitations and perspectives

On the Human AGEs web server, all of the interactive map computations occur on the client side; thus, the visualization performance is strongly dependent on the user's hardware. Moreover, Human AGEs focus solely on the visualization of endpoint genomic and archeogenomic results. In addition, it does not provide the possibility of running sophisticated bioinformatic analyses of genomic data. We also emphasize that, currently, our graph database lacks any downloadable nucleotide sequence data. However, Human AGEs were built using powerful open-source software to ensure the ease of introducing new features and expanding the graph database content.

First, we plan to expand the range of data presentation methods, including more layer types (histograms and phylogenetic trees) and data styling options (marking map layers with symbols and attributes with colored patterns). Moreover, we intend to introduce a feature for creating custom clustering regions using polygon-drawing tools or by providing an input GeoJSON file. The map utility for placing user-defined shapes, bitmaps, and text boxes together to serve as visualization annotation is also considered. Lastly, we aim to develop a feature for creating GIF animations showing the distribution of samples and their genomic traits changing over time.

Regarding the graph database, we plan to keep it up-to-date with new versions of already integrated data sources. We also plan to incorporate the kinship information determined for archaic samples, develop a separate tabular data browser for more traditional exploration, and extend the capabilities of our query builder.

## DATA AVAILABILITY

Human AGEs is freely available at https://archeogenomics.eu/. Instructions on how to use the web server were provided in the guide accessible at https://archeogenomics.eu/en/map. Web server source code is available at: https://github.com/wooksh/Human_AGEs.

## Supplementary Material

gkad428_Supplemental_File

## References

[1] Parson W. , Dür A. EMPOP–a forensic mtDNA database. Forensic Sci. Int. Genet. 2007; 1:88–92. 19083735 10.1016/j.fsigen.2007.01.018

[2] Stolarek I. , Juras A., Handschuh L., Marcinkowska-Swojak M., Philips A., Zenczak M., Dębski A., Kóčka-Krenz H., Piontek J., Kozlowski P. et al. A mosaic genetic structure of the human population living in the South Baltic region during the Iron Age. Sci. Rep. 2018; 8:2455. 29410482 10.1038/s41598-018-20705-6 PMC5802798

[3] Stolarek I. , Handschuh L., Juras A., Nowaczewska W., Kóčka-Krenz H., Michalowski A., Piontek J., Kozlowski P., Figlerowicz M. Goth migration induced changes in the matrilineal genetic structure of the central-east European population. Sci. Rep. 2019; 9:6737. 31043639 10.1038/s41598-019-43183-w PMC6494872

[4] Gretzinger J. , Sayer D., Justeau P., Altena E., Pala M., Dulias K., Edwards C.J., Jodoin S., Lacher L., Sabin S. et al. The Anglo-Saxon migration and the formation of the early English gene pool. Nature. 2022; 610:112–119. 36131019 10.1038/s41586-022-05247-2 PMC9534755

[5] Fu Q. , Posth C., Hajdinjak M., Petr M., Mallick S., Fernandes D., Furtwängler A., Haak W., Meyer M., Mittnik A. et al. The genetic history of Ice Age Europe. Nature. 2016; 534:200–205. 27135931 10.1038/nature17993 PMC4943878

[6] Haak W. , Lazaridis I., Patterson N., Rohland N., Mallick S., Llamas B., Brandt G., Nordenfelt S., Harney E., Stewardson K. et al. Massive migration from the steppe was a source for Indo-European languages in Europe. Nature. 2015; 522:207–211. 25731166 10.1038/nature14317 PMC5048219

[7] Ehler E. , Novotný J., Juras A., Chyleński M., Moravčík O., Pačes J. AmtDB: a database of ancient human mitochondrial genomes. Nucleic Acids Res. 2019; 47:D29–D32. 30247677 10.1093/nar/gky843 PMC6324066

