# Supplementary material for: Human AGEs: an interactive spatio-temporal visualization and database of human archeogenomics
Source: Nucleic Acids Res. 2023 May 22;51(W1):W269–73. doi: 10.1093/nar/gkad428 (PMC10320146; doi:10.1093/nar/gkad428)
Supplement: gkad428_Supplemental_File [file gkad428_supplemental_file.pdf]

SUPPLEMENTARY DATA

Human AGEs: an interactive spatio-temporal visualization and database of human archeogenomics

Lukasz Ciecierski [1], Ireneusz Stolarek [1], Marek Figlerowicz [1] *

[1] Institute of Bioorganic Chemistry, Polish Academy of Sciences, Poznan, Poland

* To whom correspondence should be addressed. Tel: +48 61 852 89 19;
Fax: +48 61 852 05 32; Email: marek.figlerowicz@ibch.poznan.pl

METHODS

Admixture, UMAP and PCA analyzes preparation

For the Admixture analysis the AADR v54.1 dataset was filtered as follows: all the present day samples were excluded and only the unrelated ancient individuals from ~24 000 years BP (the oldest individual is AfontovaGora2) were kept. With individuals prefiltered, pseudohaploidization of the genetic data was performed, SNP positions with MAF>5% were kept, LD pruning with plink ver. 1.90 was performed with the parameters: -indep-pairwise 200 25 0.6, and any loci genotyped in less than 1% of the samples were removed.
The same SNP filtering options were applied for the PCA calculation. PCA was calculated with smartpca software ver. 7.2. 50 PCs were calculated and the first two components were used for the visualization. Next all 50 PCs were provided to the umap R implementation ver. 0.2.10 for the two-dimensional embedding calculation with default settings.

TABLES

Table S1. Essential software used during Human AGEs web server back-end development.

Name	Version
nodejs	13.14.0
build-essential	2.0.0
npm	7.8.0
nodejs express	4.17
openlayers	6.9.0
openlayers-ext	3.2.3
openlayers-mapbox-style	6.3.2
graphql	14.6.0
neo4j-driver	4.0.2
neo4j-graphql-js	2.13.0
babel	7.9.6
webpack	5.28.0
pug.js	3.0.2
node-sass	4.13.1
deepmerge	4.2.2
apollo-server-express	2.19.0
apollo-fetch	0.7.0

Table S2. Software used during Human AGEs web server front-end development.

Name	Version
jQuery	3.3.1
jquery-easing	1.4.1
jquery-mousewheel	3.1.13
malihu-custom-scrollbar-plugin	3.1.5
popper	1.16.1
bootstrap	4.5.3
json5	2.1.3
jqueryui	1.12.1
bootstrap-select	1.13.17
flexsearch	0.7.2
jquery.isotope	3.0.6
tinycolor	1.4.2
Chart.js	3.8.0
Ion.RangeSlider	2.3.1
bootbox	5.4.0
doT	1.1.3
jquery-extendext	1.0.0
polyfill	v2
bootstrap4-toggle	3.6.1
bootstrap4-tagsinput	4.1.3
d3	7.0.0
d3-cloud	1.2.5
spectrum	1.8.1
grapick	0.1.13

seedrandom	3.0.5
jspdf	1.3.2
bootstrap-table	1.20.0
jquery-scrollintoview	1.8
tippy	5.0.3
anglepicker	1.0.1
anno.js	1.0.0
plotly	1.33.1
jQuery-QueryBuilder	2.5.2

Table S3. Dataset attribute types that are recognized from input files and supported by Human AGE visualization features.

Name	Description	Examples of use
property	Any textual non-unique attribute. Useful for archeological or anthropological properties.	sex; human development stage; burial type
tree	Values of a text attribute are nodes of a tree. Only Y and mt haplogroups are supported.	Y and mt haplogroups
proportion	Any comma separated multi-dimensional vector of real numbers, which sums up to 1.0,<br>e.g. 0.2,0.1,0.2,0.5.	admixture analysis genomic components
vector	Any comma separated 2D vector of real numbers, e.g. 0.0013,5.0.	PCA and UMAP coordinates of genomic embeddings

Table S4. Possible user input dataset content. The user can load a dataset in JSON or CSV format. Valid JSON input file must be a list of dictionaries, where each contains key-value pairs corresponding to sample's attribute name and value pairs. Valid CSV file must contain a header in the first line with sample attribute names and each next row must contain tab-separated attribute values of a single sample. Some attribute names are reserved, so any custom user-provided attribute must have a different name to be properly detected. Optional attributes can be specified in the input file with blank strings as values for samples with missing data.

Name	Description	Examples	Mandatory
sample_id	Text attribute. Must be a unique value.	female; adult; grave burial	yes
latitude	Real number describing latitude coordinate of sample's geographic position. Must be a valid number for an assumed coordinate projection.	Y and mt haplogroups	yes
longitude	Real number describing longitude coordinate of sample's geographic position. Must be a valid number for an assumed coordinate projection.	admixture analysis genomic components	yes
projection	Carthographic projection of the sample's coordinates. If none is provided, it will be EPSG:4326 as default. If provided, they will be converted to the interactive map's projection.	EPSG:4326	no
dating_from & dating_to	Sample's dating lower and upper bound. Can be either integer or text string. Preferably negative values for BC / BCE dates and positive for AD / CE. YBP (years before present) notation is not yet supported.	-1000; -1000 BC; -1000 BCE; 966; 966 AD; 966 CE	no
population_name	Any text string that indicates population name of the contemporary genomics samples.	England_BellBeaker	no

place_name	Any text string that indicates archaeological site or present-day sampling place.	Wojkowice	no
sex	Genetic or archaeological sex determination.	male/female	no
haplogroup_y & haplogroup_mt	Text attribute of root type. Its values must correspond to haplogroups present in the graph database. If not, warning will be displayed on data load and attribute’s values will be set to blank for given sample.	R1b; U8c	no
admixture_k<N>	Attribute of the proportion type. Number <N> must match attribute value’s vector length. Comma separated values.	0.2,0.1,0.2,0.5	no
admixture_k<N>: <name_1>, <name_2>,..., <name_N>	The same as above with a possibility to specify names for each of genomic components. The names will be displayed in the map legend. Comma separated values.	0.2,0.1,0.2,0.5	no
pca	Attribute of the vector type. Contains PCA embedding consisting of any two components values. Comma separated values.	0.0013,5.0	no
umap	Attribute of the vector type. Contains UMAP embedding consisting of any two components values. Comma separated values.	0.0013,5.0	no
<custom_name>	Any custom attribute without provided type name will be recognized as property type.	*Any text string*	no
<custom_name>-<type_name>	Custom attribute recognized as an attribute type provided after a single dash. An exception is tree type, which cannot be assigned to any custom attribute.	*Any value valid for specified attribute type*	no

Table S5. The comparison of the Human AGEs webserver to other similar web applications. Their web addresses are following:
AmtDB - https://amtdb.org/, EMPOP - https://empop.online/, AncientGenomes - https://ancientgenomes.com/, UMAP Openstreetmap - https://umap.openstreetmap.fr/en/map/ancient-human-dna_41837#6/51.000/2.000, PhyloGeoViz - http://phylogeoviz.org/, Haplotree - https://haplotree.info/maps/ancient_dna/
Bolded font in the table indicates unique features of the given web resource.

Name	Data content and type	Visualization customization	Can load user data	Other important features
Human AGEs	present day genomic, archeogenomic, archeological, anthropological data; mtDNA and Y-DNA haplogroups, **genome-wide UMAP/PCA analysis, genomic admixture components**, archeological cultures regions	customizable map, **customizable data appearance and map layers**	yes	interactive filtering of data in time; filtering data by multiple attributes; **attributes grouping; adjustable automatic spatial and regional clustering; presenting multiple data layers at the same time; possibility to save and restore visualization**; downloadable samples annotation; **sample data can be presented using point, piecharts, heatmaps and tag clouds**
AmtDB	archeogenomic, archeological, anthropological data; mtDNA haplogroups	customizable map	no	filtering data in time; filtering data by multiple attributes; simple automatic spatial grouping; downloadable samples annotation and **genomic data**; sample data presented only as points
EMPOP	present day genomic data; mtDNA haplogroups	no	no	**can explore samples by using mtDNA haplogroups tree**; simple automatic spatial grouping; sample data presented only as points

Name	Data content and type	Visualization customization	Can load user data	Other important features
Ancient Genomes	archeogenomic, archeological, linguistic data; mtDNA and Y-DNA haplogroups, archeological cultures regions,	data layers can be toggled on/off	no	interactive filtering of data in time; multiple layers of data; sample data presented only as points
UMAP Openstreetmap	archeogenomic data; mtDNA and Y-DNA haplogroups	customizable map	no	sample data presented only as points; simple data filtering
PhyloGeoViz	only user-provided DNA haplotype data	no	yes	sample data presented as piecharts
Haplotree	archeogenomic, archeological data; mtDNA and Y-DNA haplogroups, archeological cultures	customizable map	no	filtering of data in time; filtering data by multiple attributes; displaying summary statistics for regions

FIGURES

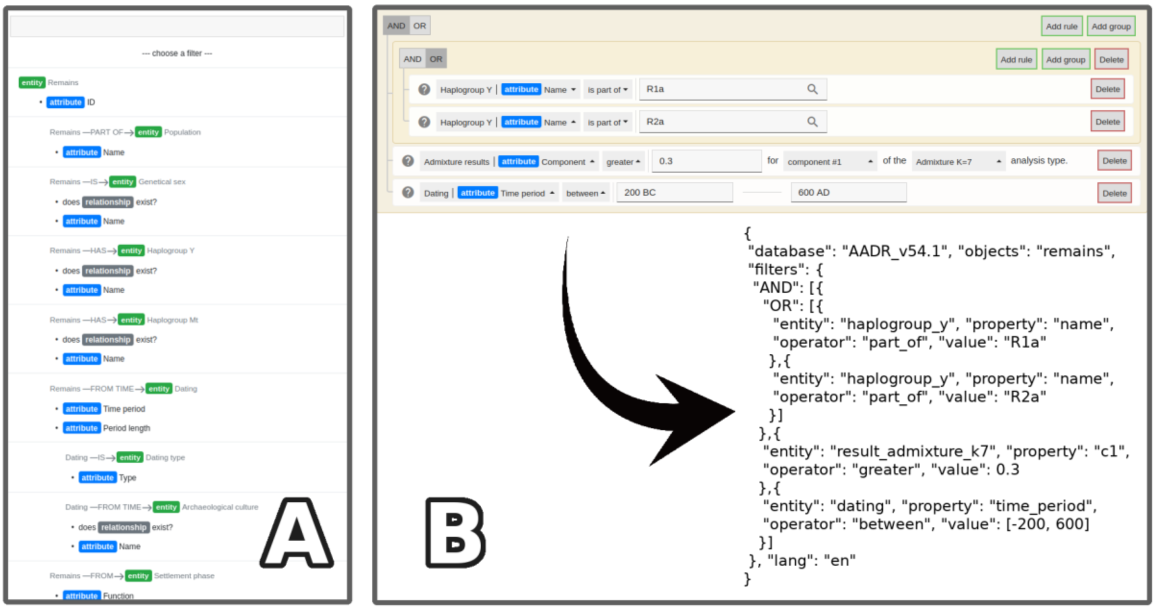

Figure S1. Query builder interface. A) Example of available query builder filters. B) Chosen data source and filters defined in query builder are translated to the JSON query string. The query string is then sent to the server and translated to the Neo4j graph database Cypher query statement.

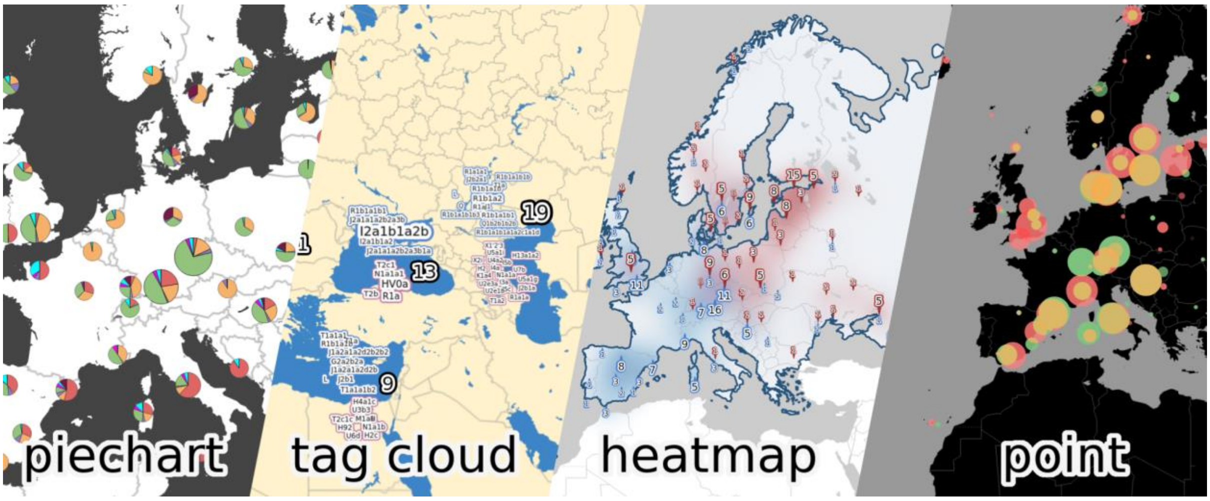

Figure S2. Main four data representation types. All of them can be used to visualize samples distribution, however piecharts and tag clouds are additionally specialized to display attribute values frequencies and cluster content.

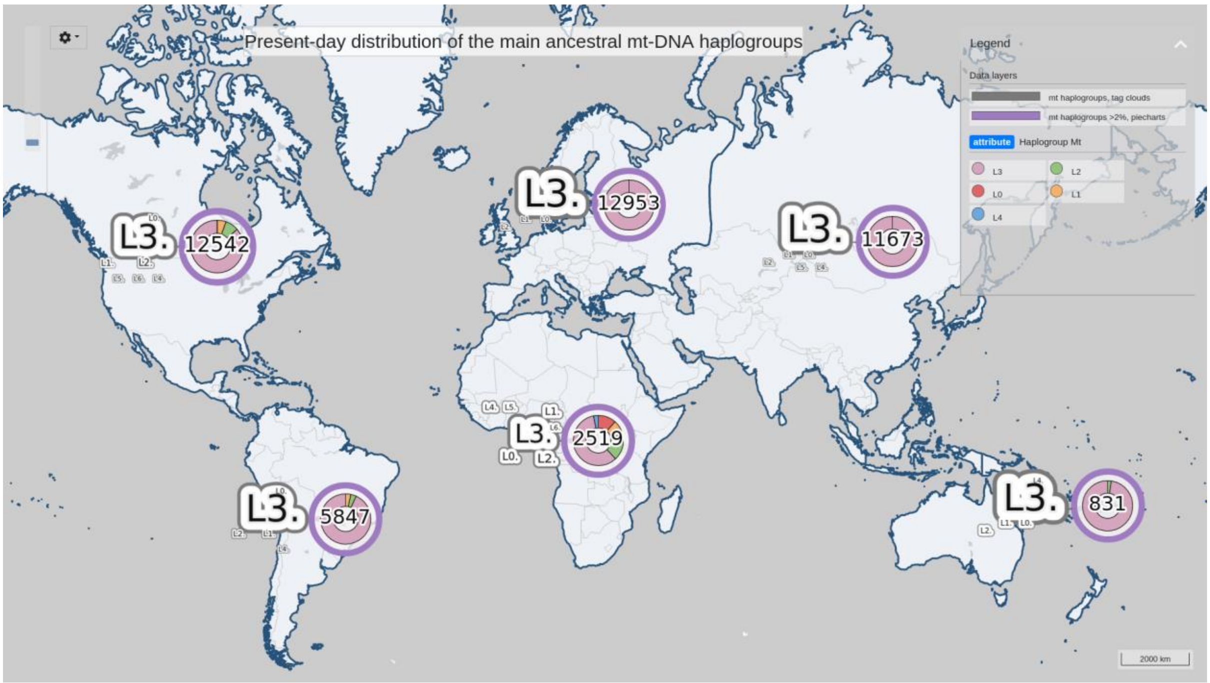

Figure S3. Main ancestral mt-DNA haplogroups distribution of EMPOP samples. The samples were clustered by continents. Piechart layer was adjusted to show haplogroups of frequency larger than 2%.
